# “Duckweed-Microbe Co-Cultivation Method” for Isolating a Wide Variety of Microbes Including Taxonomically Novel Microbes

**DOI:** 10.1264/jsme2.ME18067

**Published:** 2018-11-07

**Authors:** Yasuhiro Tanaka, Hideyuki Tamaki, Kazuya Tanaka, Erina Tozawa, Hiroaki Matsuzawa, Tadashi Toyama, Yoichi Kamagata, Kazuhiro Mori

**Affiliations:** 1 Graduate School of Life and Environmental Sciences, University of Yamanashi 4–4–37 Takeda, Kofu, Yamanashi 400–8510 Japan; 2 Bioproduction Research Institute, AIST 1–1–1 Higashi, Tsukuba, Ibaraki 305–8566 Japan; 3 Graduate School of Engineering, University of Yamanashi 4–3–11 Takeda, Kofu, Yamanashi 400–8511 Japan; 4 International Research Centre for River Basin Environment, University of Yamanashi 4–3–11 Takeda, Kofu, Yamanashi 400–8511 Japan

**Keywords:** *Armatimonadetes*, duckweed, aquatic plant, microbial isolation

## Abstract

We herein described a new microbial isolation method using the interaction between the floating aquatic plant, duckweed, and microbes. We harvested microbial cells from Japanese loosestrife roots and co-cultivated these cells with aseptic duckweed using artificial inorganic medium for the plant for four weeks. During the co-cultivation, some duckweeds were collected every week, and the roots were used for microbial isolation using a low-nutrient plate medium. As a result, diverse microbial isolates, the compositions of which differed from those of the original source (Japanese loosestrife root), were obtained when the roots of duckweed were collected after 2 weeks of cultivation. We also successfully isolated a wide variety of novel microbes, including two strains within the rarely cultivated phylum, *Armatimonadetes*. The present study shows that a duckweed-microbe co-cultivation approach together with a conventional technique (direct isolation from a microbial source) effectively obtains more diverse microbes from a sole environmental sample.

Macrophytes are plants that are widely distributed across various aquatic environments, such as rivers, ponds, and lakes, and have been classified into floating, emergent, and submergent plants in relation to their habitats. Since most of these plants have water purification abilities, these plants, particularly those in wetlands, have been used for wastewater treatments mainly targeting nitrogen and phosphorous removal (1, 2, 5, 13). Recent studies reported that microbes in the rhizospheres of macrophytes are involved in the degradation of toxic organic compounds (6, 14–17). Acting in concert with this, a number of microbial strains involved in the degradation of chemicals, *e.g.* nonylphenol (17), 4-*tert*-butylphenol (16), 3-nitrophenols (6), and bisphenol A (8), have been isolated from the rhizospheres of several aquatic plants. However, limited information is currently available on the microbial communities that interact with aquatic plant roots. Therefore, we analyzed microbes inhabiting the roots of the floating aquatic plant, *Spirodela polyrhiza* (duckweed), and four emergent plants, *Phragmites australis* (reed), *Lythrum anceps* (Japanese loosestrife), *Iris pseudacorus* (yellow iris), and *Scirpus juncoides* (tule), and the results obtained revealed that these aquatic plants harbor particularly diverse microbes, including a wide variety of novel microbes, on their roots (9, 11, 12).

We succeeded in isolating many taxonomically novel microbes, including the rarely cultivated bacterial phylum represented by *Armatimonadetes* (formerly called candidate division OP10) from the roots of aquatic plants (9, 11, 12).

We proposed the following reasons for why these results were obtained: 1) aquatic plants continuously recruit species-specific microbes, which may attach and adapt to root environments, from miscellaneous microbial communities living in their surroundings, and 2) acclimate these microbes in an easily cultivable state by supplying root exudates, the entities and functions of which have yet to be clarified. If these hypotheses are true, the co-cultivation of a microbial-free (aseptic) plant (hereafter called the “recruiter plant”) and microbes from an environmental sample (the microbial source) may provide unique microbial consortia with high affinity to the “recruiter plant”. Furthermore, it may be possible to obtain microbes that are otherwise difficult to isolate directly from the original source. In the present study, we aimed to establish whether this method, “the co-cultivation of recruiter plant and microbes”, effectively isolates unique diverse microbes including taxonomically novel microorganisms. We used aseptic duckweed as a recruiter plant and microorganisms inhabiting the roots of Japanese loosestrife, a common aquatic plant.

## Materials and Methods

### Microbial isolation

The roots of Japanese loosestrife (*L. ancep*) that grew in a pond in Yamanashi prefectural woody park (Fuefuki, Yamanashi, Japan; 35°38′23″ N, 138°40′36″ E) was used as a microbial source. Approximately 0.54 g (wet weight) of the plant roots was gently washed twice with 30 mL of sterilized Hoagland medium (14) in a 50-mL conical tube, and the roots were milled (15,000 rpm for 7 min) in 10 mL of sterilized Hoagland medium using a homogenizer (Ace HOMOGENIZER AM-1; Nihonseiki, Tokyo, Japan). The homogenate was diluted in 10-fold steps with sterilized Hoagland medium. The diluted sample (50 μL) was inoculated on a low-nutrient DTS (1/100-strength tryptic soy broth; Becton Dickinson, Franklin Lakes, NJ, USA) (pH 7.0) agar plate (9) in triplicate, and incubated at 25°C (we hereafter define this cultivation method as STD) for 29 d under dark conditions. The homogenized solution was diluted to 10% with sterilized Hoagland medium at a volume of 300 mL in a 500-mL plant culture pot. We used the duckweed, *S. polyrhiza*, in the present study as a “recruiter plant”; five aseptic plants and microbes derived from Japanese loosestrife roots were added to the plant pot with Hoagland medium, and kept in a growth chamber (6,900 lux; 16:8 h light-dark cycle) at 25°C. After the 1-d incubation, duckweed that associated with the recruited microbes was transferred into 300 mL of new sterilized Hoagland medium in a 500-mL plant pot, and cultivated for 1, 2, 3, or 4 weeks in a growth chamber with the same conditions as those described above (we hereafter defined this method as DMC [duckweed-microbe co-cultivation]-1w, -2w, -3w or -4w, respectively). A duckweed-free control (further referred to as DFC) batch was also prepared; 350 μL of the diluted root homogenate, which was similar to the water volume for the five duckweed plants, was added to 300 mL of sterilized Hoagland medium without duckweed, and cultivated for 1 week in a growth chamber. The isolation of microbes from the roots of duckweed (three plants for each batch) and the water sample was performed as follows: the plants were gently washed twice with 30 mL of sterile Hoagland medium in a 50-mL test tube, and the roots were cut off aseptically with a surgical knife. The cut roots were homogenized in 10 mL of sterilized Hoagland medium at 15,000 rpm for 5 min using the homogenizer (Ace HOMOGENIZER AM-1), and the homogenate was inoculated on DTS agar plates, as described for Japanese loosestrife roots. The water sample collected from DFC was diluted in 10-fold steps with sterile Hoagland medium, and each diluted sample (50 μL) was independently inoculated on agar (1.5%) plates in triplicate. Microbial cultivation was performed at 25°C for 29 d under dark conditions. A schematic image of the STD, DMC, and DFC cultivation methods is shown in Fig. S1.

### 16S rRNA gene-based microbial community analysis of the inoculum

Japanese loosestrife roots (0.15 g) were subjected to the extraction of total nucleic acids using the FastDNA Spin Kit for Soil (Q-Biogene, Montreal, Canada). 16S rRNA genes were amplified by PCR using two bacterial universal primers, EUB8F (18) (5′-AGAG TTTGATCMTGGCTCAG-3′: corresponding to positions 8–27 of the *Escherichia coli* 16S rRNA gene) and EUB1512R (4) (5′-ACG GYTACCTTGTTACGACTT-3′; corresponding to positions 1,492–1,512 of the *E. coli* 16S rRNA gene) from extracted DNA, as reported previously. Amplified DNA fragments were purified by the Illustra GFX PCR purification kit (GE Healthcare Life Sciences, Pittsburgh, PA, USA) and cloned into *E. coli* strain DH5α using the pT7 Blue T-vector (Merck Millipore, Burlington, MA, USA). The clone library obtained was analyzed by PCR-RFLP using two types of restriction endonucleases, *Hha*I and *Hae*III, as described in our previous study (9). The coverage (*C*) values of the clone library were calculated by the equation *C*=(1–[*n/N*])×100, where *n* and *N* are the number of unique clones and the total number of analyzed clones, respectively.

### Phylogenetic analysis of clones and isolates

The 16S rRNA gene of isolates was also amplified by colony PCR using the primers EUB8F and EUB1512, and subjected to a RFLP analysis as well as the above clone library analysis with the exception that *Msp*I was used instead of *Hae*III. The 16S rRNA gene fragments of representatives from the clonal and isolate RFLP groups were purified with the GFX PCR DNA and Gel purification kit (GE Healthcare Life Sciences), and sequenced by the method of Matsuzawa *et al.* (9). Sequence data were compared with those in the EZBioCloud database (http://www.ezbiocloud.net/eztaxon). The taxonomic classification of the sequence at the class level was conducted using the CLASSIFIER program (http://rdp.cme.msu.edu/classifier/classifier.jsp).

### Microbial diversity

The diversities of clones and isolates at the RFLP group level were evaluated by the calculation of the Shannon-Weiner index ([*H*]= −∑[*pi*][ln *pi*]) and Simpson’s reciprocal index, 1/D, where D equals ∑(*pi*) and *pi* is the proportion of groups *i* relative to the total number of RFLP groups.

### Accession numbers

The GenBank/EMBL/DDBJ accession numbers for the 16S rRNA gene sequences of clones are LC378474–LC378543, while those of the isolates are LC378717–LC378797 and LC382241.

## Results and Discussion

### 16S rRNA gene clonal analysis of the microbial source

**Fig. 1:**
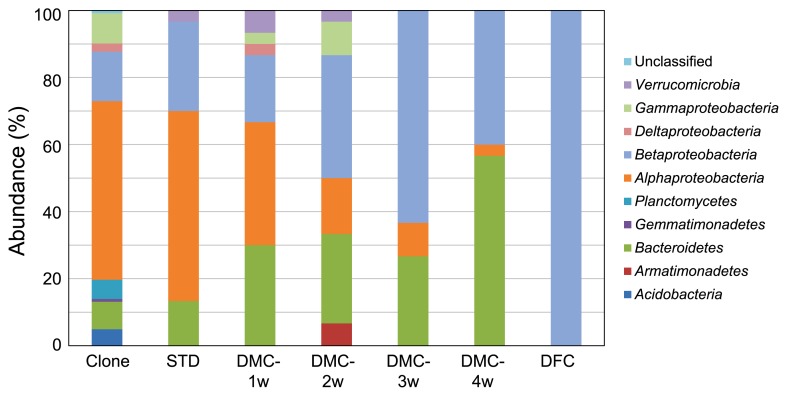
Phylogenetic distribution of 16S rRNA gene clones from the original microbial source (Japanese loosestrife roots) and isolates obtained by STD, DMC, and DFC methods.

**Fig. 2:**
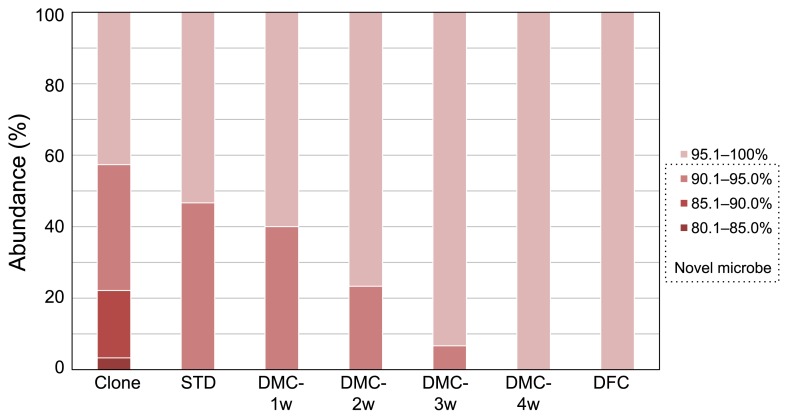
Taxonomic novelty of clones and isolates. Similarity percentages of the 16S rRNA gene between the clones recovered from the roots of Japanese loosestrife (Clone) or isolates (STD, DMC-1w, DMC-2w, DMC-3w, DMC-4w, and DFC) and their closest authentic species are shown.

We previously demonstrated that Japanese loosestrife harbors a wide variety of novel microbes on its roots with a relatively high yield (76% of all analyzed clones) (11). Therefore, we selected the roots of this plant as the microbial source in this study. To confirm whether the roots of Japanese loosestrife used in the present study also harbor a plethora of taxonomically novel microbes, as shown in our previous study, we analyzed their microbial composition using a culture-independent method based on the 16S rRNA gene sequence. The PCR-RFLP analysis grouped 122 clones into 70 phylotypes (Table S1), and the coverage value was estimated to be 63.1%. The phylogenetic analysis based on the 16S rRNA gene sequence showed that the clones comprised 8 bacterial taxonomic divisions and unclassified microbes (Fig. 1), and the most predominant class was *Alphaproteobacteria*, which is consistent with our previous findings (11). The taxonomic novelty of the clones was evaluated using the criterion that sequences have less than 95% similarity to the 16S rRNA gene of any known bacterial species, and sequences with the value indicated above were defined as those from phylogenetically novel microbes. Based on this criterion, 57% of clones were judged to be novel microbes (Fig. 2). Although this score is slightly lower than that in our previous study (11), it still maintains a level higher than 50%, indicating that the root sample used in the present study was effective as a source for isolating taxonomically novel microbes.

### Microbial isolation

Thirty colonies were randomly selected from each DTS plate, and applied to the 16S rRNA gene-based PCR-RFLP analysis. A total of 180 isolates were grouped into 61 phylotypes consisting of 19 for STD, 23 for DMC-1w, 17 for DMC-2w, 11 for DMC-3w, 7 for DMC-4w, and 3 for DFC methods (Table S2). Based on RFLP phylotypes, the diversity of the isolates was evaluated by Shannon-Weiner and Simpson’s reciprocal indices (Table 1). In our previous study, indices for the microbial isolates of Japanese loosestrife roots were higher than those of pond water collected from the surroundings of the plant, and, thus, a wide variety of microbes, including many novel bacterial species, were easily isolated from the roots (11). Since the scores in the STD method were similar to those in our previous study (2.96 for the Shannon-Weiner index and 13.56 for Simpson’s reciprocal index) (11) (Table 1), the high diversity of the isolates from Japanese loosestrife was also confirmed in this study. In the isolates from the DMC method, equivalent levels of indices to those by the STD method were maintained after up to 2 weeks of co-cultivation, whereas these scores were markedly reduced after 3 weeks (Table 1). In natural duckweed plants living in water environments, various types of microbes are continuously supplied from the surrounding water, and, thus, microbial diversity in the roots may be maintained at a relatively high level. Actually, a wide variety of microbes were isolated from the roots of wild duckweed in our previous study (9). In contrast, in the DMC method, the supply of microbes to aseptic duckweed plants only occurred once in the inoculation step; therefore, the microbes that inhabited the duckweed root environment were very likely to dominate during co-cultivation. However, the lowest diversity of isolates was observed in the DFC method (DFC cultivation), strongly indicating that the richness of diversity in DMC-1w and DMC-2w was dependent on the presence of the duckweed plant body.

### Taxonomic analysis of isolates

To identify the taxonomic position of the isolates, the partial sequence of the 16S rRNA gene from a representative strain of each RFLP group was elucidated and compared with those in the EZBioCloud database. Table S2 shows the most closely related species of the isolates. *Alphaproteobacteria* was the most abundant bacterial class in the isolates obtained by the STD method (Fig. 1). In microbial isolation using the DMC method, the taxonomic compositions of the isolates markedly differed from those by the STD method: in the early phase (1 week) of co-cultivation (DMC), the members of three bacterial groups, *Alphaproteobacteria*, *Betaproteobacteria*, and *Bacteroidetes*, were the main constituents of the population of isolates; however, the abundance of *Betaproteobacteria* gradually increased until 3 weeks, and then *Bacteroidetes* markedly increased and was the dominant bacterial group in 4 weeks (Fig. 1). All isolates obtained by the DFC method belonged to *Betaproteobacteria* (Fig. 1). At the family level of classification, 8 bacterial families were obtained by STD methods, whereas 17, 12, 8, 5, and 2 bacterial families were found in the isolates from the DMC-1w, -2w, -3w, -4w, and DFC methods, respectively (Table 2). Within the bacterial families obtained by the DMC method, 12 families for 1 week, 7 for 2 weeks, 5 for 3 weeks, and 3 for 4 weeks of co-cultivation were not found in the isolates from the STD method, indicating that the present method (DMC) effectively provided diverse microbial isolates, the compositions of which differed from those of the original microbial source.

On the other hand, since all bacterial families from DMC-3w and -4w were also included in the isolates of DMC-1w or -2w, and microbial diversities after 3 weeks of co-cultivation were significantly reduced (Table 1), the combined use of DMC, particularly up to 2 weeks of co-cultivation, and STD methods was effective for capturing more diverse microbial species from aquatic plant roots.

The taxonomical novelty of isolates was also evaluated based on the same definition used in a molecular-based microbial community analysis of the roots of Japanese loosestrife. Using the STD method, phylogenetically novel microbes were isolated with a high yield (47%). The DFC method did not recover any novel microbes, whereas the DMC method (co-cultivation with duckweed) yielded 40 and 23% of novel microbes after 1 and 2 weeks of cultivation, respectively, while none of the novel microbes were isolated after 3 weeks of co-cultivation (Fig. 2). Furthermore, we successfully isolated two taxonomically novel microbes, strains LA-DMC-2W6 and LA-DMC-2W26, belonging to the phylum *Armatimonadetes* by DMC-2w. The phylum *Armatimonadetes* is a rarely cultivated microbial group, and only 4 strains (one strain of *Armatimonas rosea*, two strains of *Chthonomonas calidirosea*, and one strain of *Fimbriimonas ginsengisoli*) have been isolated to date (3, 7, 10). These results and data regarding microbial diversity suggest that confining the co-cultivation period to 2 weeks in the DMC method is better for isolating a wide variety of novel microorganisms.

In the present study, we targeted only the roots of Japanese loosestrife, which have a good track record for diverse and novel microbes as bacterial isolation sources. We are currently isolating microbes from more common samples, such as river water, pond water, and soil, using the DMC method to confirm the applicability of this method. In addition, we are planning further research to verify our scenario that duckweed recruits microbes that adapt to the root environment from miscellaneous microbial communities in the original source and acclimates them to an easily cultivable state: 1) a culture-independent analysis of microbial community changes in the roots of duckweed during duckweed-microbe co-cultivation, and 2) an examination of the effects of duckweed on the cultivability of microbes obtained by the DMC method. The results obtained will be reported elsewhere.

## Supplementary Material



## Figures and Tables

**Table 1:** Diversity indices for isolates

Diversity indices	STD	DMC				DFC

		1 week	2 weeks	3 weeks	4 weeks	
Richness*	19 (30)	23 (30)	17 (30)	11 (30)	7 (30)	3 (30)
Shannon-Weiner index	2.79	3.03	2.70	2.06	1.39	0.53
Simpson’s reciprocal index	14.1	18.0	13.2	5.84	2.76	0.58

* Richness indicates the number of isolate phylotypes.

The number of isolates obtained in this study was shown in parentheses.

**Table 2:** Taxonomic classification of clones and isolates at the family level

Phylum	Class	Order	Family	Number of clones*	Number of Isolates					

					STD	DMC-1 week	DMC-2 weeks	DMC-3 weeks	DMC-4 weeks	DFC
*Acidobacteria*	Acidobacteria GP3	Unclassified	Unclassified	4						
	Acidobacteria GP6	Unclassified	Unclassified	1						
	*Acidimicrobiales*	*Acidimicrobiaceae*	Unclassified	1						

*Armatimonadetes*	Armatimonadetes GP5	Unclassified	Unclassified				2			

*Bacteroidetes*	*Chitinophagia*	*Chitinophagales*	*Chitinophagaceae*	6	4	6	4	2		
			Unclassified	1						
	*Cytophagia*	*Cytophagales*	*Cytophagaceae*			1	2	1	17	
	*Flavobacteriia*	*Flavobacteriales*	*Flavobacteriaceae*			2	2	5		
	*Saprospiria*	*Saprospirales*	*Saprospiraceae*	1						
			Unclassified	2						

*Gemmatimonadetes*	*Gemmatimonadetes*	*Gemmatimonadales*	*Gemmatimonadaceae*	1						

*Planctomycetes*	*Planctomycetia*	*Planctomycetales*	*Planctomycetaceae*	7						

*Proteobacteria*	*Alphaproteobacteria*	*Caulobacterales*	*Caulobacteraceae*	2	1	1	3			
		*Rhizobiales*	*Beijerinckiaceae*	2						
			*Bradyrhizobiaceae*	3		1	1		1	
			*Hyphomicrobiaceae*	15						
			*Rhizobiaceae*	5		3		3		
			Rhizobiales incertae sedis		2		1			
			Unclassified	16	9	1				
		*Rhodospirillales*	*Rhodospirillaceae*			1				
		*Sphingomonadales*	*Sphingomonadaceae*	9	5	3				
		Unclassified	Unclassified	13		1				

	*Betaproteobacteria*	*Burkholderiales*	*Alcaligenaceae*	3						
			Burkholderiales Genera incertae sedis	6	7	2	3	3	6	29
			*Comamonadaceae*		1		4	14	5	
			*Oxalobacteraceae*			2		1		1
			Unclassified			1				
		*Methylophilales*	*Methylophilaceae*	4		1	4	1	1	
			Unclassified	1						
		Unclassified	Unclassified	4						

	*Deltaproteobacteria*	*Bdellovibrionales*	*Bdellovibrionaceae*	2						
		*Myxococcales*	*Myxococcaceae*			1				
		Unclassified	Unclassified	1						

	*Gammaproteobacteria*	*Methylococcales*	Unclassified	1						
		*Nevskiales*	*Sinobacteraceae*	1						
		*Pseudomonadales*	*Pseudomonadaceae*			1	3			
		Unclassified	Unclassified	9						

*Verrucomicrobia*	*Opitutae*	*Opitutales*	*Opitutaceae*		1					
	*Verrucomicrobiae*	*Verrucomicrobiales*	*Verrucomicrobiaceae*			2	1			

Unclassified	Unclassified	Unclassified	Unclassified	1						

Total number of clones or isolates				122	30	30	30	30	30	30

* 16S rRNA gene clones obtained from the roots of Japanese loosestrife
