# Supplementary material for: “Duckweed-Microbe Co-Cultivation Method” for Isolating a Wide Variety of Microbes Including Taxonomically Novel Microbes
Source: Microbes Environ. 2018 Nov 7;33(4):402–6. doi: 10.1264/jsme2.ME18067 (PMC6308005; doi:10.1264/jsme2.ME18067)
Supplement: Supplementary file 1 [file 33_402_s1.pdf]

Table S1. Phylogenetic afiliations of 16S rRNA gene clones obtained from the root of Japnese loosestrife

RFLP Group	Representative clone	Clone number	Authentic species (Accession No.)	Identity (%)	Phylum (Class)	Length (bp)
1	JL-C5	5	*Hyphomicrobium facile* subsp. *tolerans* (Y14311)	92.90	*Proteobacteria* (Alpha)	704
2	JL-C59	1	*Povalibacter uvarum* (AB548216)	90.64	*Proteobacteria* (Gamma)	716
3	JL-C39	3	*Ferruginibacter lapsinanis* (FJ177532)	96.08	*Bacteroidetes*	740
4	JL-C49	9	*Labrys methylaminiphilus* (AY766152)	92.87	*Proteobacteria* (Alpha)	631
5	JL-C41	7	*Tepidamorphus gemmatus* (GU187912)	91.16	*Proteobacteria* (Alpha)	735
6	JL-C45	2	*Hyphomicrobium hollandicum* (Y14303)	91.59	*Proteobacteria* (Alpha)	750
7	JL-C56	3	*Methylophilus leisingeri* (AF250333)	95.58	*Proteobacteria* (Beta)	636
8	JL-C65	1	*Methylotenera versatilis* (CP002056)	97.01	*Proteobacteria* (Beta)	737
9	JL-C63	1	*Rubrivivax gelatinosus* (D16213)	97.76	*Proteobacteria* (Beta)	715
10	JL-C9	3	*Azohydromonas lata* (D88007)	97.80	*Proteobacteria* (Beta)	636
11	JL-C98	2	*Rhizobacter fulvus* (AB245356)	99.03	*Proteobacteria* (Beta)	720
12	JL-C71	1	*Nitrosospira lacus* (CAUA01000011)	90.51	*Proteobacteria* (Beta)	728
13	JL-C73	2	*Paludibaculum fermentans* (KJ461654)	90.36	*Acidobacteria*	726
14	JL-C74	1	*Blastobacter capsulatus* (X73042)	98.20	*Proteobacteria* (Alpha)	724
15	JL-C7	4	*Phreatobacter oligotrophus* (HE616165)	95.60	*Proteobacteria* (Alpha)	637
16	JL-C11	3	*Novosphingobium lentum* (AJ303009)	98.57	*Proteobacteria* (Alpha)	631
17	JL-C104	1	*Bdellovibrio bacteriovorus* (BX842601)	93.89	*Proteobacteria* (Delta)	720
18	JL-C12	2	*Bradyrhizobium lablabi* (GU433448)	100	*Proteobacteria* (Alpha)	638
19	JL-C83	2	*Hyphomicrobium facile* subsp. *tolerans* (Y14311)	96.50	*Proteobacteria* (Alpha)	742
20	JL-C6	2	*Hyphomicrobium zavarzinii* (KB911255)	94.56	*Proteobacteria* (Alpha)	735
21	JL-C91	1	*Sphingorhabdus rigui* (HQ436492)	95.99	*Proteobacteria* (Alpha)	724
22	JL-C69	2	*Steroidobacter denitrificans* (EF605262)	88.35	*Proteobacteria* (Gamma)	721
23	JL-C38	5	*Thioalbus denitrificans* (EU837269)	88.85	*Proteobacteria* (Gamma)	628
24	JL-C87	1	*Gimesia maris* (ABCE01000043)	86.55	*Planctomycetes*	715
25	JL-C93	2	*Ensifer kostiensis* (AM181748)	97.66	*Proteobacteria* (Alpha)	725
26	JL-C92	1	*Aquincola tertiaricarbonis* (DQ656489)	96.39	*Proteobacteria* (Beta)	721
27	JL-C97	1	*Ferruginibacter lapsinanis* (FJ177532)	96.80	*Bacteroidetes*	719
28	JL-C17	2	*Rhizobium giardinii* (ARBG01000149)	97.82	*Proteobacteria* (Alpha)	734
29	JL-C114	1	*Rhizobium endophyticum* (EU867317)	99.71	*Proteobacteria* (Alpha)	684
30	JL-C48	1	*Methylobacterium marchantiae* (FJ157976)	90.25	*Proteobacteria* (Alpha)	636
31	JL-C110	2	*Portibacter lacus* (AB675658)	87.22	*Bacteroidetes*	713
32	JL-C111	1	*Filomicrobium fusiforme* (Y14313)	94.58	*Proteobacteria* (Alpha)	720
33	JL-C106	1	*Aciditerrimonas ferrireducens* (AB517669)	89.66	*Actinobacteria*	725
34	JL-C112	1	*Hyphomicrobium facile* subsp. *tolerans* (Y14311)	97.79	*Proteobacteria* (Alpha)	725
35	JL-C117	1	*Parasegetibacter terrae* (KJ634465)	91.69	*Bacteroidetes*	698
36	JL-C119	1	*Thermogutta hypogea* (KC867695)	85.01	*Planctomycetes*	727
37	JL-C120	1	*Gemmatimonas phototrophica* (AUXF01000006)	90.73	*Gemmatimonadetes*	701
38	JL-C29	2	*Sphingomonas asaccharolytica* (Y09639)	99.20	*Proteobacteria* (Alpha)	628
39	JL-C115	1	*Gemmata obscuriglobus* (ABGO01000325)	86.99	*Planctomycetes*	707
40	JL-C1	4	*Hyphomicrobium facile* subsp. *tolerans* (Y14311)	97.23	*Proteobacteria* (Alpha)	721
41	JL-C3	1	*Blastopirellula marina* (AANZ01000021)	87.18	*Planctomycetes*	734
42	JL-C4	1	*Altererythrobacter namhicola* (FJ935793)	87.47	*Proteobacteria* (Alpha)	718
43	JL-C13	2	*Alsobacter metallidurans* (AB231946)	96.95	*Proteobacteria* (Alpha)	623
44	JL-C18	1	*Ochrobactrum rhizosphaerae* (AM490632)	87.62	*Proteobacteria* (Alpha)	719
45	JL-C20	1	*Asticcacaulis excentricus* (CP002395)	97.30	*Proteobacteria* (Alpha)	629
46	JL-C54	2	*Sphingomonas suberifaciens* (FJ796422)	97.33	*Proteobacteria* (Alpha)	750
47	JL-C16	1	*Caldicoprobacter algeriensis* (GU216701)	82.20	*Acidobacteria*	720
48	JL-C23	1	*Leptothrix mobilis* (X97071)	98.74	*Proteobacteria* (Beta)	633
49	JL-C24	3	*Blastopirellula marina* (AANZ01000021)	88.23	*Planctomycetes*	624
50	JL-C31	1	*Caulobacter daechungensis* (JX861096)	95.91	*Proteobacteria* (Alpha)	635
51	JL-C32	1	*Arachidicoccus rhizosphaerae* (EU672808)	89.42	*Bacteroidetes*	737
52	JL-C8	2	*Thioprofundum lithotrophicum* (AB468957)	89.30	*Proteobacteria* (Gamma)	626
53	JL-C14	1	*Haliscomenobacter hydrossis* (CP002691)	84.65	*Bacteroidetes*	749
54	JL-C15	1	*Methyloferula stellata* (ARWA01000001)	96.90	*Proteobacteria* (Alpha)	741
55	JL-C21	1	*Methylosoma difficile* (DQ119050)	92.90	*Proteobacteria* (Gamma)	635
56	JL-C19	1	*Flavitalea populi* (HM130561)	95.66	*Bacteroidetes*	760

Table S1. Phylogenetic afiliations of 16S rRNA gene clones obtained from the root of Japnese loosestrife (*continued*)

RFLP Group	Representative clone	Clone number	Authentic species (Accession No.)	Identity (%)	Phylum (Class)	Length (bp)
57	JL-C22	1	*Methylophilus leisingeri* (AF250333)	94.70	*Proteobacteria* (Beta)	741
58	JL-C26	1	*Denitratisoma oestradiolicum* (AY879297)	91.27	*Proteobacteria* (Beta)	733
59	JL-C27	1	*Tepidisphaera mucosa* (KM036168)	84.85	*Planctomycetes*	628
60	JL-C30	1	*Methylocapsa aurea* (JQKO01000009)	97.70	*Proteobacteria* (Alpha)	739
61	JL-C37	1	*Anaeromyxobacter dehalogenans* (CP001359)	84.58	*Proteobacteria* (Delta)	720
62	JL-C51	1	*Methylovirgula ligni* (FM252034)	94.13	*Proteobacteria* (Alpha)	698
63	JL-C52	1	*Rubrivivax gelatinosus* (D16213)	97.20	*Proteobacteria* (Beta)	715
64	JL-C64	1	*Novosphingobium lentum* (AJ303009)	95.98	*Proteobacteria* (Alpha)	697
65	JL-C72	1	*Paludibaculum fermentans* (KJ461654)	90.69	*Acidobacteria*	698
66	JL-C79	1	*Nitrosospira lacus* (CAUA01000011)	91.93	*Proteobacteria* (Beta)	715
67	JL-C77	1	*Amorphus orientalis* (FJ998414)	93.79	*Proteobacteria* (Alpha)	698
68	JL-C88	1	*Bdellovibrio bacteriovorus* (BX842601)	87.32	*Proteobacteria* (Delta)	696
69	JL-C90	1	*Paludibaculum fermentans* (KJ461654)	91.11	*Acidobacteria*	686
70	JL-C113	1	*Zoogloea oleivorans* (KF667502)	90.11	*Proteobacteria* (Beta)	708
		122				

The RFLP group whose sequences showed less than 95% identity with those of authentic species were underlined.

Table S2. Isolates and their related authentic species based on the sequence of 16S rRNA gene

RFLP group	STD		DMC-1 week		DMC-2 weeks		DMC-3 weeks		DMC-4 weeks		DFC		Authentic species (Accession No.)	Identity (%)	Phylum (Class)	Length (bp)
	Representative strain	Strain No.	Representative strain	Strain No.	Representative strain	Strain No.	Representative strain	Strain No.	Representative strain	Strain No.	Representative strain	Strain No.				
1	LA-STD1	1							LA-DMC-4W1	4			*Pelomonas saccharophila* (AB021407)	99.34	*Proteobacteria* (Beta)	760
2	LA-STD2	1	LA-DMC-1W25	1									*Chelatovorus multitrophus* (EF457243)	91.31	*Proteobacteria* (Alpha)	771
3	LA-STD4	3											*Devosia albogilva* (EF433460)	90.22	*Proteobacteria* (Alpha)	767
4	LA-STD5	4											*Novosphingobium acidiphilum* (D16147)	97.90	*Proteobacteria* (Alpha)	762
5	LA-STD6	3											*Tepidamorphus gemmatus* (GU187912)	90.96	*Proteobacteria* (Alpha)	774
6	LA-STD8	1											*Hyphomicrobium hollandicum* (Y14303)	90.64	*Proteobacteria* (Alpha)	781
7	LA-STD9	2											*Niastella jeongjuensis* (DQ244076)	95.06	*Bacteroidetes*	770
8	LA-STD10	1											*Rhizobacter dauci* (AB297965)	98.93	*Proteobacteria* (Beta)	755
9	LA-STD11	3											*Leptothrix mobilis* (X97071)	96.03	*Proteobacteria* (Beta)	755
10	LA-STD17	1											*Novosphingobium lindaniclasticum* (ATHL01000125)	97.80	*Proteobacteria* (Alpha)	772
11	LA-STD18	1											*Hyphomicrobium hollandicum* (Y14303)	90.68	*Proteobacteria* (Alpha)	763
12	LA-STD19	1											*Ideonella dechloratans* (D16213)	96.99	*Proteobacteria* (Beta)	768
13	LA-STD20	1											*Rubrivivax gelatinosus* (D16213)	97.55	*Proteobacteria* (Beta)	776
14	LA-STD22	1											*Asticcacaulis benevestituss* (AM087199)	97.81	*Proteobacteria* (Alpha)	778
15	LA-STD23	2											*Hyphomicrobium hollandicum* (Y14303)	91.49	*Proteobacteria* (Alpha)	777
16	LA-STD24	1											*Flavitalea populi* (HM130561)	93.89	*Bacteroidetes*	737
17	LA-STD25	1											*Opitutus terrae* (CP001032)	93.03	*Verrucomicrobia*	746
18	LA-STD26	1											*Niastella populi* (EU877262)	92.96	*Bacteroidetes*	753
19	LA-STD28	1											*Aquincola tertiaricarbonis* (DQ656489)	96.27	*Proteobacteria* (Beta)	750
20			LA-DMC-1W1	1	LA-DMC-2W12	3			LA-DMC-4W15	1			*Methylophilus rhizosphaerae* (EU194887)	98.29	*Proteobacteria* (Beta)	819
21			LA-DMC-1W2	1									*Rhizobium sullae* (Y10170)	97.95	*Proteobacteria* (Alpha)	783
22			LA-DMC-1W4	2									*Novosphingobium sediminicola* (FJ177534)	97.02	*Proteobacteria* (Alpha)	739
23			LA-DMC-1W5	1	LA-DMC-2W1	2	LA-DMC-3W9	4					*Flavobacterium fontis* (JN873147)	98.21	*Bacteroidetes*	781
24			LA-DMC-1W7	2									*Rhizobium rosettiformans* (EU781656)	98.49	*Proteobacteria* (Alpha)	793
25			LA-DMC-1W8	1									*Azospirillum doebereinerae* (AJ238567)	96.46	*Proteobacteria* (Alpha)	831
26			LA-DMC-1W9	2									*Heliimonas saccharivorans* (JX458466)	94.68	*Bacteroidetes*	790
27			LA-DMC-1W12	1					LA-DMC-4W17	3			*Piscinibacter aquaticus* (DQ664244)	99.23	*Proteobacteria* (Beta)	782
28			LA-DMC-1W13	1									*Flavobacterium aquidurense* (AM177392)	97.48	*Bacteroidetes*	794
29			LA-DMC-1W14	1			LA-DMC-3W29	2					*Aquincola tertiaricarbonis* (DQ656489)	97.96	*Proteobacteria* (Beta)	792
30			LA-DMC-1W17	1	LA-DMC-2W9	1							*Asticcacaulis benevestidus* (AQWM01000073)	98.72	*Proteobacteria* (Alpha)	785
31			LA-DMC-1W15	1									*Cystobacter gracilis* (DQ768110)	91.74	*Proteobacteria* (Delta)	873
32			LA-DMC-1W19	1									*Phreatobacter oligotrophus* (HE616165)	93.55	*Proteobacteria* (Alpha)	807
33			LA-DMC-1W20	1									*Herbaspirillum aquaticum* (FJ267649)	99.62	*Proteobacteria* (Beta)	801
34			LA-DMC-1W21	4	LA-DMC-2W22	4	LA-DMC-3W17	2					*Sediminibacterium salmoneum* (KI866530)	94.03	*Bacteroidetes*	737
35			LA-DMC-1W22	1	LA-DMC-2W27	3							*Pseudomonas taiwanensis* (EU103629)	97.96	*Proteobacteria* (Gamma)	786
36			LA-DMC-1W23	1									*Paucimonas lemoignei* (X92554)	92.26	*Proteobacteria* (Beta)	776
37			LA-DMC-1W26	2	LA-DMC-2W21	1							*Luteolibacter pohnpeiensis* (KP101281)	94.98	*Verrucomicrobia*	737
38			LA-DMC-1W27	1	LA-DMC-2W15	1	LA-DMC-3W23	1	LA-DMC-4W29	17			*Flectobacillus roseus* (EU420062)	99.59	*Bacteroidetes*	772

Table S2. Isolates and their related authentic species based on the sequence of 16S rRNA gene (*continued*)

RFLP group	STD		DMC-1 week		DMC-2 weeks		DMC-3 weeks		DMC-4 weeks		DFC		Authentic species (Accession No.)	Identity (%)	Phylum (Class)	Length (bp)
	Representative strain	Strain No.	Representative strain	Strain No.	Representative strain	Strain No.	Representative strain	Strain No.	Representative strain	Strain No.	Representative strain	Strain No.				
39			LA-DMC-1W11	1									*Novosphingobium aromaticivorans* (CP000248)	97.83	*Proteobacteria* (Alpha)	739
40			LA-DMC-1W24	1									*Pseudoduganella violaceinigra* (AY376163)	98.24	*Proteobacteria* (Beta)	738
41			LA-DMC-1W3	1									*Bradyrhizobium erythrophlei* (KF114645)	99.73	*Proteobacteria* (Alpha)	740
42					LA-DMC-2W11	1							*Ideonella azotifigens* (EU542576)	98.12	*Proteobacteria* (Beta)	747
43					LA-DMC-2W17	3							*Pelomonas saccharophila* (AB021407)	99.59	*Proteobacteria* (Beta)	735
44					LA-DMC-2W19	1							*Methylophilus flavus* (FJ872108)	98.37	*Proteobacteria* (Beta)	738
45					LA-DMC-2W20	2							*Phenylobacterium conjunctum* (AJ227767)	97.97	*Proteobacteria* (Alpha)	738
46					LA-DMC-2W23	1							*Alsobacter metallidurans* (AB231946)	96.34	*Proteobacteria* (Alpha)	738
47					LA-DMC-2W26	2							*Fimbriimonas ginsengisoli* (CP002763)	92.11	*Armatimonadetes*	1292
48					LA-DMC-2W4	1							*Flectobacillus roseus* (EU420062)	99.59	*Bacteroidetes*	737
49					LA-DMC-2W5	1			LA-DMC-4W5	1			*Bradyrhizobium erythrophlei* (KF114645)	99.87	*Proteobacteria* (Alpha)	739
50					LA-DMC-2W8	2							*Rhizobacter dauci* (AB297965)	99.04	*Proteobacteria* (Beta)	736
51					LA-DMC-2W28	1	LA-DMC-3W26	4					*Pelomonas saccharophila* (AB021407)	98.12	*Proteobacteria* (Beta)	750
52							LA-DMC-3W1	1					*Flavobacterium fontis* (JN873147)	98.37	*Bacteroidetes*	738
53							LA-DMC-3W4	3					*Rhizobium aggregatum* (X73041)	99.05	*Proteobacteria* (Alpha)	738
54							LA-DMC-3W12	1					*Methylophilus methylotrophus* (AB193724)	98.93	*Proteobacteria* (Beta)	839
55							LA-DMC-3W11	10	LA-DMC-4W28	1			*Pelomonas saccharophila* (AB021407)	100	*Proteobacteria* (Beta)	735
56							LA-DMC-3W14	1					*Massilia namucuonensis* (JF799985)	97.25	*Proteobacteria* (Beta)	836
57							LA-DMC-3W19	1					*Ideonella azotifigens* (EU542576)	98.08	*Proteobacteria* (Beta)	837
58									LA-DMC-4W6	3			*Ideonella dechloratans* (X72724)	97.20	*Proteobacteria* (Beta)	833
59											LA-DFC-4W4	4	*Aquincola tertiaricarbonis* (DQ656489)	96.95	*Proteobacteria* (Beta)	822
60											LA-DFC-4W15	1	*Herbaspirillum seropedicae* (Y10146)	95.54	*Proteobacteria* (Beta)	740
61											LA-DFC-4W30	25	*Aquitalea magnusonii* (DQ018117)	99.39	*Proteobacteria* (Beta)	819
Total		30		30		30		30		30		30				

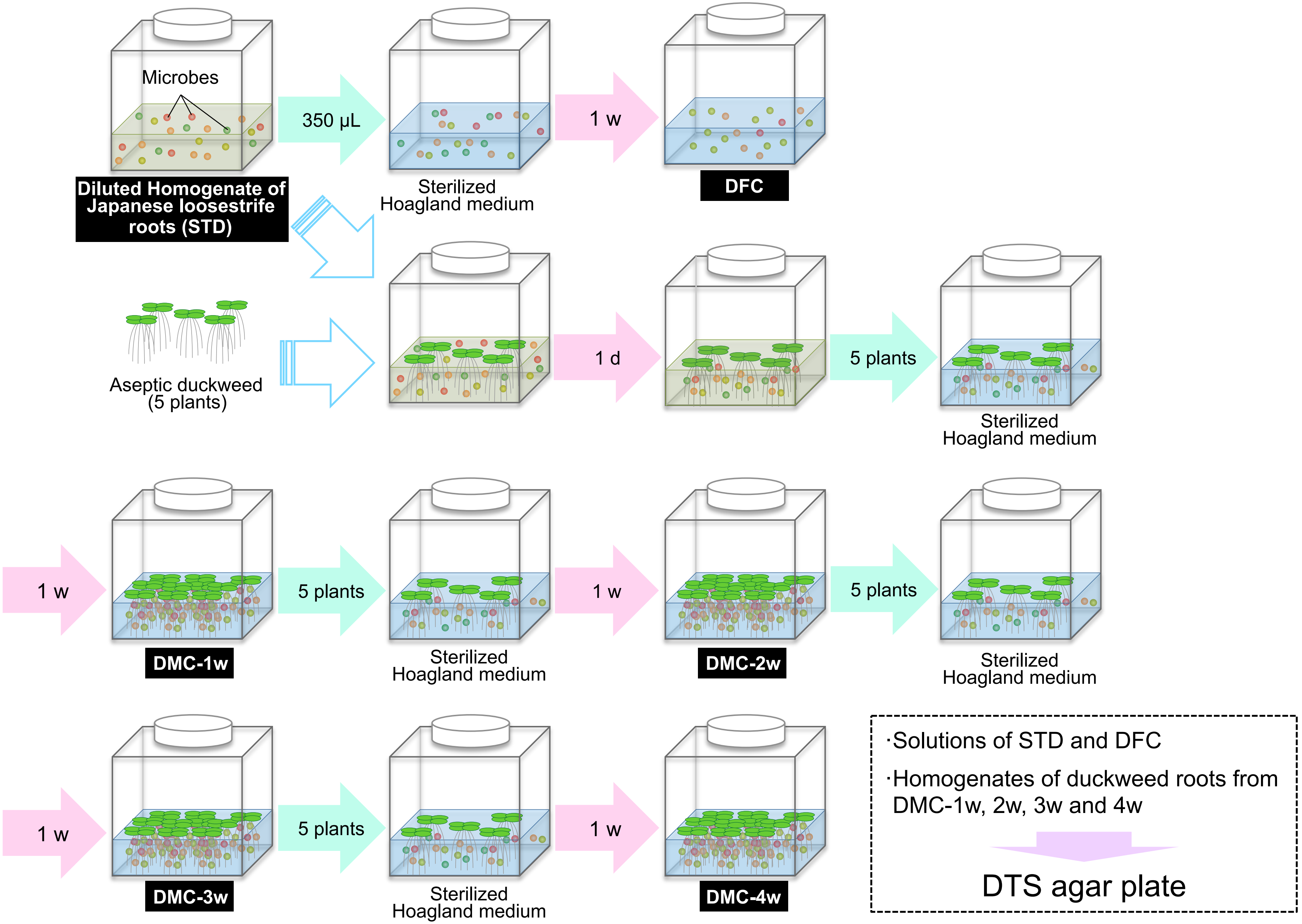

Fig. S1
Tanaka et al.

Fig. S1. Schematic image of microbial isolation methods used in the present study.
